# Stress and anxiety among healthcare workers during the COVID-19 pandemic in russia

**DOI:** 10.1192/j.eurpsy.2021.285

**Published:** 2021-08-13

**Authors:** E. Mosolova, D. Sosin, S. Mosolov

**Affiliations:** 1 Faculty Of Basic Medicine, Lomonosov Moscow State University, Moscow, Russian Federation; 2 Department Of Psychiatry, Russian Medical Academy of Continuous Professional Education, Moscow, Russian Federation; 3 Department Of Pharmacotherapy, Moscow Research Institute of Psychiatry, Moscow, Russian Federation

**Keywords:** Anxiety, COVID-19, SAVE-9, GAD-7

## Abstract

**Introduction:**

Mental health of medical workers treating patients with COVID-19 is an issue of increasing concern worldwide, since previous epidemics have shown high levels of anxiety and stress in front-line healthcare professionals. The available data on stress and anxiety symptoms among healthcare workers during the COVID-19 are relatively limited and have not been evaluated in Russia yet.

**Objectives:**

To evaluate stress and anxiety symptoms among healthcare workers directly involved in the diagnosis and treatment of patients with COVID-19 during the peak of disease outbreak in Russia.

**Methods:**

The study was a cross-sectional hospital-based anonymous on-line survey in May 2020 of 1,090 healthcare workers practicing treatment of patients with COVID-19. Stress and anxiety symptoms were assessed using the Russian versions of Stress and Anxiety to Viral Epidemic scale (SAVE-9) and Generalized Anxiety Disorder (GAD-7) scales. Logistic regression analysis was performed to determine the influence of different variables.

**Results:**

The median scores on the GAD-7 and SAVE-9 were 5 and 14, respectively. 49.1% respondents had moderate and 21.9% had severe anxiety according to SAVE-9. 12.3% had severe anxiety, 13.2% had moderate according to GAD-7. Female gender and younger age were associated with higher level of anxiety according to regression model.

**Conclusions:**

Our study has shown that healthcare workers in Russia practicing treatment of patients with COVID-19 reported high rates of stress and anxiety similar to other countries. Female gender, younger age and being a physician were associated with higher levels of anxiety. These results demonstrate the importance of supportive programs for health care workers fighting COVID-19.

**Disclosure:**

No significant relationships.

